# Smartphone App for Prehospital ECG Transmission in ST-Elevation Myocardial Infarction Activation: Protocol for a Mixed Methods Study

**DOI:** 10.2196/55506

**Published:** 2024-09-06

**Authors:** Hassan Mir, Katelyn J Cullen, Karen Mosleh, Rafi Setrak, Sanjit Jolly, Michael Tsang, Gregory Rutledge, Quazi Ibrahim, Michelle Welsford, Mathew Mercuri, JD Schwalm, Madhu K Natarajan

**Affiliations:** 1 University of Ottawa Heart Institute University of Ottawa Ottawa, ON Canada; 2 Ottawa Hospital Research Institute University of Ottawa Ottawa, ON Canada; 3 Hamilton Health Sciences McMaster University Hamilton, ON Canada; 4 Niagara Health St. Catharines, ON Canada; 5 Population Health Research Institute Hamilton, ON Canada; 6 William Osler Health System Brampton, ON Canada; 7 Institute of Health Policy, Management and Evaluation University of Toronto Toronto, ON Canada

**Keywords:** ST-elevation myocardial infarction, m-health, cardiac systems of care, knowledge mobilization, digital health, smartphone technology, technology, STEMI, Canada, implementation, mobile phone

## Abstract

**Background:**

Timely diagnosis and treatment for ST-elevation myocardial infarction (STEMI) requires a coordinated response from multiple providers. Rapid intervention is key to reducing mortality and morbidity. Activation of the cardiac catheterization laboratory may occur through verbal communication and may also involve the secure sharing of electrocardiographic images between frontline health care providers and interventional cardiologists. To improve this response, we developed a quick, easy-to-use, privacy-compliant smartphone app, that is SMART AMI-ACS (Strategic Management of Acute Reperfusion and Therapies in Acute Myocardial Infarction Acute Coronary Syndromes), for real-time verbal communication and sharing of electrocardiographic images among health care providers in Ontario, Canada. The app further provides information about diagnosis, management, and risk calculators for patients presenting with acute coronary syndrome.

**Objective:**

This study aims to integrate the app into workflow processes to improve communication for STEMI activation, resulting in decreased treatment times, improved patient outcomes, and reduced unnecessary catheterization laboratory activation and transfer.

**Methods:**

Implementation of the app will be guided by the Reach, Effectiveness, Acceptability, Implementation, and Maintenance (RE-AIM) framework to measure impact. The study will use quantitative registry data already being collected through the SMART AMI project (STEMI registry), the use of the SMART AMI app, and quantitative and qualitative survey data from physicians. Survey questions will be based on the Consolidated Framework for Implementation Research. Descriptive quantitative analysis and thematic qualitative analysis of survey results will be conducted. Continuous variables will be described using either mean and SD or median and IQR values at pre- and postintervention periods by the study sites. Categorical variables, such as false activation, will be described as frequencies (percentages). For each outcome, an interrupted time series regression model will be fitted to evaluate the impact of the app.

**Results:**

The primary outcomes of this study include the usability, acceptability, and functionality of the app for physicians. This will be measured using electronic surveys to identify barriers and facilitators to app use. Other key outcomes will measure the implementation of the app by reviewing the timing-of-care intervals, false “avoidable” catheterization laboratory activation rates, and uptake and use of the app by physicians. Prospective evaluation will be conducted between April 1, 2022, and March 31, 2023. However, for the timing- and accuracy-of-care outcomes, registry data will be compared from January 1, 2019, to March 31, 2023. Data analysis is expected to be completed in Fall 2024, with the completion of a paper for publication anticipated by the end of 2024.

**Conclusions:**

Smartphone technology is well integrated into clinical practice and widely accessible. The proposed solution being tested is secure and leverages the accessibility of smartphones. Emergency medicine physicians can use this app to quickly, securely, and accurately transmit information ensuring faster and more appropriate decision-making for STEMI activation.

**Trial Registration:**

ClinicalTrials.gov NCT05290389; https://clinicaltrials.gov/study/NCT05290389

**International Registered Report Identifier (IRRID):**

DERR1-10.2196/55506

## Introduction

ST-segment elevation myocardial infarction (STEMI) requires efficient communication and collaboration to ensure timely care. Clinical practice guidelines recommend patients who present to a percutaneous coronary intervention (PCI)–capable center should receive PCI within 90 minutes, and those presenting to a non–PCI-capable center should receive PCI within 120 minutes [1]. Those receiving care beyond these time thresholds have an increased risk of mortality, reinfarction, congestive heart failure, and rehospitalization compared with those who receive care within these time thresholds [2-4]. However, achieving timely care can be challenging, especially for patients in rural and remote areas with limited resources.

Despite guideline recommendations, patients fail to receive timely care [5]. There are over 7000 STEMI cases in Ontario every year [5], the majority of which present to non–PCI-capable centers [6]. The Ontario Ministry of Health has set a target that more than 75% of patients should receive care within the time thresholds mentioned above. However, only 49% of patients presenting to a PCI-capable hospital and 44% of patients presenting to a non–PCI-capable center received care within these thresholds [5]. This may be due to several factors, including local geography, weather constraints, delay in diagnosis, delay in transfer, or delay in management by the STEMI team [7]. The COVID-19 pandemic also had a significant impact on the delivery of STEMI care. Prepandemic (2019), 42% of patients presenting to a PCI-capable center in Ontario received care within 90 minutes. This reduced to 28% during the pandemic (2020). Similarly, those presenting to non–PCI-capable centers noted a decline from 47% of patients receiving care within 120 minutes down to 37% during the pandemic [8]. This highlights an important and ongoing need.

The current process for STEMI activation in the Hamilton, Ontario, Canada, health care region typically relies on the emergency medicine (EM) physician making a diagnosis of STEMI and contacting the interventional cardiologist (IC) on call. The suspected STEMI case is reviewed by telephone, which is coordinated through a STEMI hotline. A 12-lead electrocardiography (ECG) is essential in the diagnosis of a STEMI, and, as per the 2021 American Heart Association policy statement, should be communicated with the IC before activating the STEMI team and accepting the patient for transfer [9]. The approved process for ECG transmission is by a fax machine and, while secure, this method has its challenges. For example, it requires both the referring EM physician and the IC to have easy access to a functioning fax machine. This is not always the case, especially for the IC, who is often away from the hospital while on call. Where the ECG cannot be transmitted to the IC, the risk of inappropriate transfer to the catheterization laboratory can occur, potentially leading to false or “avoidable” activation, where the team is activated when the patient does not, in fact, have a STEMI or evidence of significant coronary artery disease [10]. That may lead to inefficient use of scarce resources, downstream delays for other patients, and unnecessary risk of procedure for the patient transferred [11].

An alternative to fax transmission is texting a photo of the ECG from the EM physician to the IC, using a mobile phone. However, standard texting software is not privacy-compliant and may lead to a breach of personal health information. Some providers have explored the use of a secure smartphone app as a strategy to transfer ECG data while overcoming privacy concerns. There are limited data showing the effectiveness and acceptability of this technology. For example, a retrospective, before-and-after trial using an app for communication at a center in the United States was also able to demonstrate a 22% improvement in the timing of care [12]. Similarly, a study performed in China demonstrated a reduction in the time to activate the STEMI team, the timing of intervention, and an increase in the proportion of patients meeting the guideline-recommended timing for care [13]. Furthermore, in New York, ECG images from a sample of STEMI cases were managed through an app, leading to decreased treatment times [14,15]. While these studies are important to demonstrate the feasibility of the use of an app, they were small and were performed in health care system contexts very different from what exists across Canada. Furthermore, the apps used may not conform to Canadian-specific privacy requirements for smartphone technology. As such, it is less likely that these apps would be approved for use in our local setting.

Recognizing the need for improved communication with the challenges described, a team of local clinicians created a fast, secure, easy-to-use, and privacy-compliant smartphone app, that is, SMART AMI-ACS (Strategic Management of Acute Reperfusion and Therapies in Acute Myocardial Infarction Acute Coronary Syndromes), which allows real-time ECG review, thus allowing rapid decision-making. The app is designed to be used for all cases where a STEMI is suspected by EM physicians following a diagnostic ECG. CorHealth Ontario, a provincial organization that oversees the quality of cardiac care across Ontario, recently released recommendations supporting the need for enhanced communication for STEMI activation during the COVID-19 pandemic [16]. Initial data from one hospital group in our region (Niagara Health), on the acceptability and user experience among EM and IC physicians, shows promise [17], but is insufficient to promote the technology as an evidence-based innovation for care. By expanding the use of the app and conducting this implementation research study, we hope to further close the knowledge gap on a potentially useful method for optimizing the timeliness of care in our region [18-20].

The SMART AMI-ACS app research study will enable the assessment of the app’s implementation and impact across 14 hospitals and urgent care centers, with a population of 1.4 million people [21]. We will work in collaboration with the Centre for Evidence-Based Implementation (CEBI) at Hamilton Health Sciences (HHS) to conduct pre- and postimplementation surveys that assess the barriers and facilitators to implementing the app, and the acceptability and sustainability of continued app use. The findings of this project will be shared with our knowledge user group with the goal of scaling our experience to implement the app across the remaining STEMI systems in Ontario and beyond. We will also explore opportunities to expand the use of the app to the prehospital ambulance services in this region. The overall aim of this mixed methods evaluation is to assess the feasibility and effectiveness of integrating a smartphone app in the treatment of suspected STEMI.

## Methods

### Overview

We propose a multicenter, mixed methods observational study focused on EM physicians who care for patients presenting to our 14 regional partner hospitals (a list of participating hospitals can be found in Multimedia Appendix 1) with suspected STEMI. The study will use a pre-post design to evaluate the implementation and use of a clinical intervention, the SMART AMI-ACS app. This evaluation will be supported by a quantitative assessment of STEMI registry data that are already collected through the SMART AMI project at HHS, data based on app uptake and use, and quantitative and qualitative data from participating physicians’ completion of the pre- and postimplementation surveys. No new patient data will need to be collected for the purpose of this study. Objectives and outcomes for this study will be guided by the use of the Reach, Effectiveness, Acceptability, Implementation, Maintenance (RE-AIM) framework, an evaluation framework for the implementation and assessment of health interventions [22].

### Clinical Intervention

All local EM and IC physicians will be invited to use the app to communicate, transmit the ECG images, and activate the STEMI team, regardless of participation in this research study. We plan to use the app as the primary means of STEMI activation in our health region. The app enables transmission of up to 3 ECG images, which can be reviewed immediately by the IC (screenshots of the app can be found in Multimedia Appendix 2). The app further provides information about diagnosis, management, and risk calculators for patients presenting with acute coronary syndrome [1]. Full security testing, including penetration testing, has been completed as part of an initial pilot study [17].

### Study Population and Recruitment

The study will take place at the HHS general site (HGH), a larger quaternary-care cardiac center, and the 13 referring emergency departments. EM physicians working at partner emergency departments included in this study will receive email invitations to join the research study. Participation will involve providing feedback about the use of the app between 6 and 8 months after app implementation. All EM physicians working at one of the referring centers will be eligible to participate.

For evaluation of the impact of the app on timing and appropriateness of care, we will access data on a consecutive sample of patients experiencing STEMI referred from the partner emergency departments to HGH during the period of study entered into the regional STEMI database, between January 1, 2019, and March 31, 2023.

### Sample Size

We estimate that approximately 184 EM physicians are working at the referral centers in our region. As this is primarily a descriptive study examining the barriers and facilitators to the implementation of the app, we have not performed a formal sample size calculation. Based on our experience in an initial pilot study in Niagara Health, we are confident that a sufficient number of EM physicians will participate by downloading the app and providing feedback about the use of the app through a survey.

There are approximately 800 patients with STEMI per year, transferred and treated in the HGH cardiac catheterization lab. As we will be running this study over the course of 1 year, we expect to review aggregate patient-level data on approximately 800 patients with STEMI in the STEMI database. We anticipate that this is a sufficient number for regression modeling using an interrupted time series (ITS) approach.

### Analysis

Quantitative and qualitative analysis of survey results will be conducted by the research team, and adjustments will be made to the app and its implementation based on the feedback received. Survey results will be reviewed to generate themes around barriers and facilitators to app use, as well as user satisfaction, usability, and feasibility for wider distribution using Microsoft Excel and NVivo 12 (Lumivero).

Descriptive and inferential statistics will be used for quantitative data derived from the STEMI registry and physician surveys. Continuous variables will be described using either mean with SD or median and IQR (25th and 75th percentiles) at pre- and postintervention periods by the study sites. Categorical variables, such as false or “avoidable” activation, will be described as frequencies (percentages). Unadjusted comparisons of the outcomes between pre- and post-app implementation periods will be performed using *t* tests (means), Mann-Whitney *U* tests (medians), or chi-square or Fisher exact tests (if expected cell counts are less than 5 for categorical variables). For each outcome, an ITS regression model will be fitted to evaluate the impact of the app besides any underlying trend, after adjusting for potential confounders, patient demographics, and clinical characteristics, as well as variables such as days of the week, times of day, and seasons. For a continuous outcome, an ITS regression model of the following form will be fitted: *Y_i_* = *β*_0_ + *β*_1_*T_i_* + *β*_2_*X_i_* + *β*_3_*T_i_X_i_* + *β*_4_Confounder_1_*_i_* + … + *β_p_*Confounder*_pi_* + *ε_i_*, where, for the *i*^th^ patient, *Y_i_* is the continuous outcome, *T*_i_ is the time elapsed since the study started, *X_i_* is a dummy variable representing pre (*X_i_*=0) and post (*X_i_*=1) app periods, and *ε_i_* is the random error that follows a normal distribution. Here, *β*_1_ is the adjusted average change per unit time in the outcome in pre-app period (pre-app trend/slope), *β*_2_ is the adjusted average level change in the outcome following the app, and *β*_3_ is the slope change following the app. For a categorical outcome (yes or no), a log-binomial regression model will be fitted. All the variables considered for the linear model will also be considered for this model. A *P* value .05 will be considered statistically significant. Statistical analyses will be performed using Stata 13.1 (StataCorp) and SAS 9.4 (SAS Institute) software.

### Ethical Considerations

This study is approved by the Hamilton Integrated Research Ethics Board (#13643). Consent will be implied if the EM physician decides to submit information to the IC through the app. Participating EM physicians can withdraw by not using the app to transmit ECGs and instead reverting to the current practice of communication with the IC. EM physicians will be required to read information about the app’s privacy and required to click “Accept” before app use.

Consent to provide feedback will be obtained from physicians using a question at the beginning of the surveys. Participants will be notified that they can withdraw by exiting the survey without submitting it. As survey responses will be anonymous, physicians will not be able to withdraw their data after survey submission. The intervention (use of the app) represents a process change and is noninvasive with minimal risk. Individual patient consent will not be required, as patient data reviewed for this project are already being collected as standard practice through the STEMI registry to improve quality-of-care metrics.

### Objectives and Outcomes

The RE-AIM framework will be used to guide our evaluation of the app [22].

#### Objective 1

Reach means ensuring the broad availability of the app to all emergency departments and physicians in the region with 80% (240/300) of EM physicians signing up for the app. First, the number of full-time EM physicians who have downloaded the app is divided by the total number of full-time EM physicians in the sample (data from the Amazon Web Services Portal). Second, the number of EM physicians who have used the app to transmit and discuss at least 1 case (data from the Amazon Web Services Portal).

#### Objective 2

Effectiveness means assessment of any changes in evidence-based quality-of-care metrics for STEMI care, which includes (1) time from diagnosis to ambulance departure, (2) number of false positive activations, and (3) door-to-balloon time. First, timing-of-care intervals (including door-to-balloon time) for every STEMI activation. These data are already routinely collected by the STEMI registry for CorHealth auditing; therefore, there are no new data to collect. These include, but are not limited to, parameters, that are (1) EM registration to time of first ECG (reported on the ECG), (2) time of first ECG to time of EM physician assessment (reported on the patient chart), (3) time from EM physician assessment to call to the STEMI hotline (documented on the operator record), (4) time of the first call to the STEMI hotline to the time at which the IC made the decision to activate the STEMI team (documented on the operator record), (5) the time of STEMI team activation to the time for transfer to the cardiac catheterization lab at HGH (time stamp on the paramedic transport records), and (6) the time of arrival HGH to the first intervention (time stamp on the cardiac catheterization lab records).

Second, false positive activation, which is, cases where the patient with a presumed STEMI is transferred for PCI but found to have minimal to no coronary artery disease on angiography. These numbers will be divided by the total number of STEMI cases to determine false activation rates. These outcomes will be measured using data collected as part of the STEMI registry and SMART AMI program. This will be subdivided into (1) the number of cases where the patient was noted to have ST-elevation or ECG criteria for STEMI, and (2) the number of cases where the patient was not noted to have ST-elevation or ECG criteria for STEMI.

#### Objective 3

Acceptability means the assessment of the functionality of the app in real-life applications. Pre- and postimplementation surveys will assess the acceptability and usability of the app and feedback on the app following use. Questions will be based on selected domains of the Consolidated Framework for Implementation Research [23].

#### Objective 4

Implementation means encouraging routine use of the app across the region. The goal was to have 80% (240/300) of eligible STEMI activations use the app (excluding those cases presenting directly from emergency medical services or the emergency department of the HGH) to measure sustainability. These outcomes will be measured using data collected as part of the STEMI registry and SMART AMI program, which are (1) number of STEMIs activated through the app divided by the total number of STEMI activations and (2) number of STEMI activations that did not occur through the app.

#### Objective 5

Maintenance means sustainability of the adoption and metrics over a 1-year period after implementation. Some factors of maintenance include (1) measuring patient-level outcomes monthly to evaluate the sustainability of effect. This will be measured using data collected as part of the STEMI registry and SMART AMI program, and (2) obtaining physician survey data from 6 to 8 months postintervention. This will be collected through a feedback survey hosted on REDCap (Research Electronic Data Capture; Vanderbilt University).

### Data Collection and Management

As stated above, patient data will be acquired from the regional STEMI registry as part of the SMART AMI program. Patient data from the SMART AMI program are collected and deidentified by a research assistant at HHS, a community of hospitals in the Southwestern region of Ontario, from which the participating hospitals in this study reside. These deidentified patient data are shared with CorHealth, a provincial oversight body, for measuring quality and stored in an encrypted and password-protected server at HHS. A study coordinator (and postgraduate clinical fellow) will periodically audit a proportion of cases and ECGs to ensure accuracy in transcription. No protected health information will be entered into the app, as ECGs are recognized by the receiving IC according to the name of the sending physician and the time stamp.

Participating EM physicians will be invited to provide feedback about their experience with the app through a survey administered using the REDCap application [24]. Invitations will be sent by email from local physician chiefs or by research project staff approximately 6 to 8 months after implementation of the app. Participants will have 4 weeks to complete the survey, with email reminders sent as required. Survey data will be anonymous but will be associated with a specific hospital site. Survey questions will be based in part on selected domains of the Consolidated Framework for Implementation Research [23], with the goal of identifying additional barriers and facilitators to uptake and any improvements to the app’s content and implementation process. Use of the app will be tracked using Google Data Analytics for Firebase.

From a technical standpoint, the app is available on the Apple App Store and Google Play Store. It has undergone multiple user-interface expert assessments and optimization, as well as privacy testing. The app is encrypted and secure as assessed by Niagara Health and an external third-party penetration test (CyberHunter Solutions, Inc). The app is small (15-20 MB) and fast without failures in transmissions over the 12 months of the pilot study. A team of expert information technology consultants is available to onboard new physicians or solve any potential technical issues that arise with the app, and updates can be made immediately. For health care providers, the app is accessible, quick, easy to use, reliable, and secure. Data are not stored on the physician’s smartphone. However, the IC app can access the secure cloud server to access the ECGs, thus enabling real-time decision-making.

Data are encrypted and transmitted to a secure server, which stays within Ontario, Canada. The app has strict password requirements. As the IC app can access personal health information, it automatically logs out the user if the app is idle for more than 15 minutes. Data are also automatically deleted from the server every 7 days, and the data are no longer available beyond this time frame unless downloaded by the clinical and research team. This is done to reduce the risk and severity of a privacy breach.

## Results

Funding for this study began in April 2021. Preimplementation survey data were collected in the Fall of 2021 and app implementation data collection began in April 2022 and was completed in March 2023. By the end of March 2023, 84% (253/300) of physicians had downloaded the app. The analysis is anticipated to be completed by Fall 2024.

## Discussion

### Principal Findings

The delivery of timely and appropriate care is crucial for patients with STEMI, as blocked coronary arteries need immediate intervention to restore blood flow [1]. The app being evaluated in this study is designed to help with the communication and ECG transmission between EM and IC physicians so that patients can be transferred for care quicker than current practices allow. In addition, the app offers up-to-date educational resources that allow physicians to follow current standards of care regarding the optimal choice and delivery of STEMI reperfusion strategies [1].

The 1-year implementation study period will allow for user testing and feedback on the app, which will be gathered through qualitative assessment and used for app improvement in future iterations. Furthermore, results and evaluation of effectiveness may inform the expansion of the app intervention to local ambulance services and other health regions in the province.

### Dissemination Plans

Research findings will be shared with our knowledge user partners: CorHealth Ontario, Regional Emergency Services Steering Committee, and Regional Partner hospitals and emergency medical services paramedics. In addition, the findings will be shared through typical academic channels, such as conference presentations, regional rounds, and peer-reviewed journal publications. We will also leverage social media networks in cardiology and EM. Finally, we plan to distribute findings through department communication networks at each of the partner hospitals. The regional STEMI program (SMART AMI) has developed a strong network of collaboration with the 13 referring hospitals within our health region over the past 10 years that includes the development of a mechanism for feedback of STEMI quality-of-care outcomes to partner hospitals and referring clinicians. We will partner with local program leads to roll in the smartphone app as part of the STEMI care process.

### Limitations

There are several limitations that are important to highlight. First, in order to use the app, access to a reliable internet connection is needed; however, we do not expect this to be a challenge in EM departments where users can connect to their hospital Wi-Fi. This may be a greater challenge in the future when implemented among paramedics in the community, especially in rural and remote communities. The user’s smartphone operating system must also be kept up to date, which is often done automatically. If not done and there are issues in downloading or using the app, our team will provide support to address the issue. Users will need to be capable of using mobile phone technology to capture ECG image photos. Although we expect all users to know how to use the camera on their smartphone, there may be a variety in their skills of acquiring high-quality ECG images to enable accurate diagnosis. To understand this, we will be conducting a structured analysis of the ECG quality and whether it is sufficient to make a diagnosis. For security reasons, users of the IC version of the app are logged out after 15 minutes of inactivity; therefore, password recollection may be a challenge. To address this, a biometric authentication feature was added to enable immediate login based on the user’s facial recognition. The data on timing and accuracy of care are retrospective and may be prone to errors or missing data. However, a full-time staff is assigned to collect this information and ensure accuracy based on detailed chart reviews for each case. As such, missing data are expected to be rare in this study. To minimize bias in the feedback survey results, all eligible physician participants will be invited to complete the survey, and participants will be made aware that the results will be anonymous.

### Conclusions

Smartphone technology is well integrated into clinical practice and widely accessible. The proposed solution being tested is secure and leverages the accessibility of smartphones. EM physicians can use this app to quickly, securely, and accurately transmit information ensuring faster and more appropriate decision-making for transfers.

## Appendix

Multimedia Appendix 1 List of participating hospitals.

Multimedia Appendix 2 Screenshots of SMART AMI-ACS (Strategic Management of Acute Reperfusion and Therapies in Acute Myocardial Infarction Acute Coronary Syndromes) app.

## Abbreviations

CEBI: Centre for Evidence-Based Implementation
ECG: electrocardiography
EM: emergency medicine
Fax: facsimile
HGH: Hamilton Health Sciences General Site
HHS: Hamilton Health Sciences
IC: interventional cardiologist
ITS: interrupted time series
PCI: percutaneous coronary intervention
RE-AIM: Reach, Effectiveness, Acceptability, Implementation, Maintenance
REDCap: Research Electronic Data Capture
SMART AMI-ACS: Strategic Management of Acute Reperfusion and Therapies in Acute Myocardial Infarction Acute Coronary Syndromes
STEMI: ST-elevation myocardial infarction
