# Supplementary material for: Smartphone App for Prehospital ECG Transmission in ST-Elevation Myocardial Infarction Activation: Protocol for a Mixed Methods Study
Source: JMIR Res Protoc. 2024 Sep 6;13:e55506. doi: 10.2196/55506 (PMC11415716; doi:10.2196/55506)
Supplement: Multimedia Appendix 1 [file resprot_v13i1e55506_app1.docx]

List of participating hospitals:

- Brantford General Hospital
- Douglas Memorial Hospital
- Greater Niagara General Hospital
- Haldimand War Memorial Hospital
- Hamilton General Hospital
- Joseph Brant Hospital
- Juravinski Hospital
- Norfolk General Hospital
- Port Colborne Hospital
- St. Catharines General Hospital
- St. Joseph’s Healthcare Hamilton
- Welland Hospital
- West Haldimand General Hospital
- West Lincoln Memorial Hospital
