# Supplementary material for: Smartphone App for Prehospital ECG Transmission in ST-Elevation Myocardial Infarction Activation: Protocol for a Mixed Methods Study
Source: JMIR Res Protoc. 2024 Sep 6;13:e55506. doi: 10.2196/55506 (PMC11415716; doi:10.2196/55506)
Supplement: Multimedia Appendix 2 [file resprot_v13i1e55506_app2.docx]

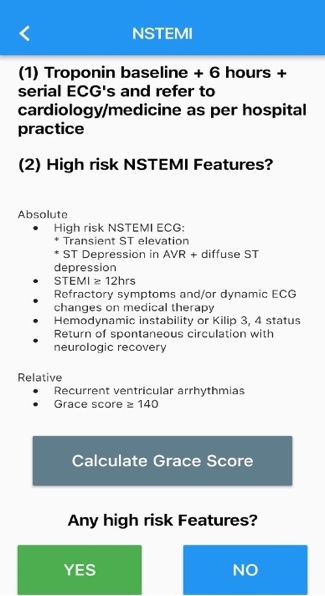

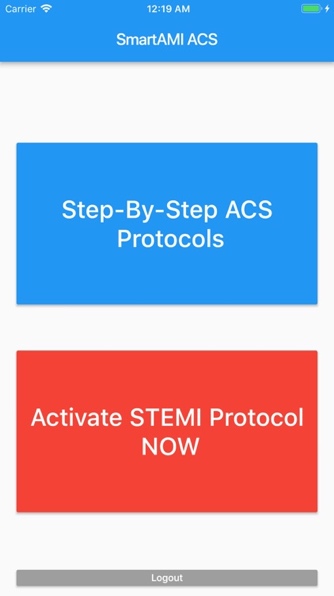

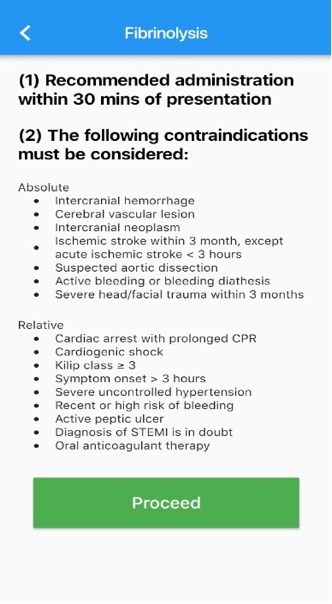

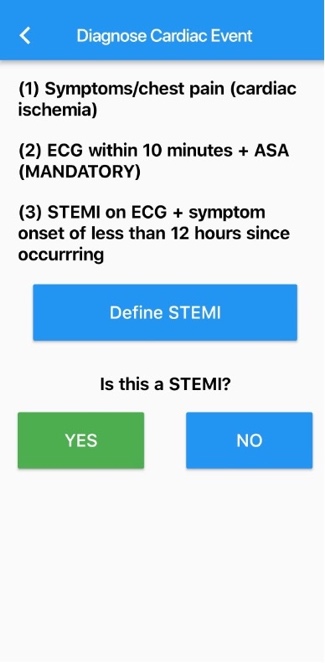

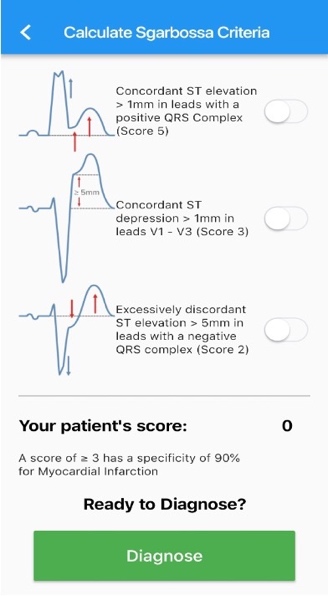

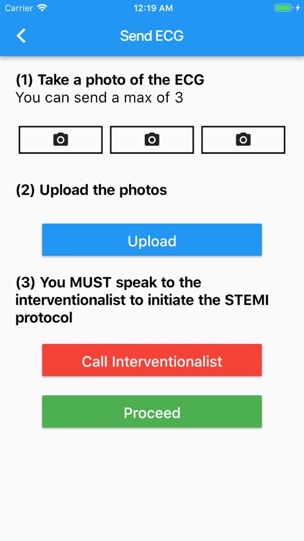

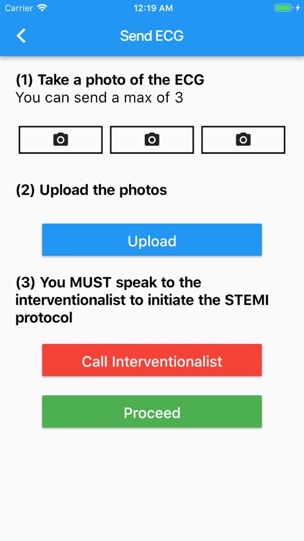

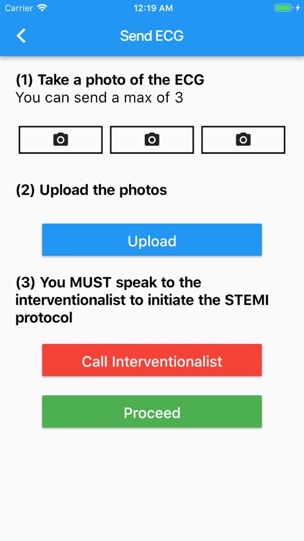


3. Select “Call interventionalist” to initiate the STEMI protocol. *ECGs will **not** be reviewed without a phone call*

2. Select “Upload”

1. Take a photo of the ECG including the patient’s name
